# Differences in the Role of HDACs 4 and 5 in the Modulation of Processes Regulating MAFbx and MuRF1 Expression during Muscle Unloading

**DOI:** 10.3390/ijms21134815

**Published:** 2020-07-07

**Authors:** Ekaterina P. Mochalova, Svetlana P. Belova, Tatiana Y. Kostrominova, Boris S. Shenkman, Tatiana L. Nemirovskaya

**Affiliations:** 1 Institute of Biomedical Problems, RAS, 123007 Moscow, Russia; mochalova_ekaterina@lenta.ru (E.P.M.); swetbell@mail.ru (S.P.B.); bshenkman@mail.ru (B.S.S.); 2 Department of Anatomy, Cell Biology and Physiology, Indiana University School of Medicine-Northwest, Gary, IN 46408, USA; tkostrom@iun.edu

**Keywords:** muscle unloading, HDACs 4 and 5, MYOG, MuRF1, MAFbx

## Abstract

Unloading leads to skeletal muscle atrophy via the upregulation of MuRF-1 and MAFbx E3-ligases expression. Reportedly, histone deacetylases (HDACs) 4 and 5 may regulate the expression of MuRF1 and MAFbx. To examine the HDAC-dependent mechanisms involved in the control of E3-ubiquitin ligases expression at the early stages of muscle unloading we used HDACs 4 and 5 inhibitor LMK-235 and HDAC 4 inhibitor Tasqinimod (Tq). Male Wistar rats were divided into four groups (eight rats per group): nontreated control (C), three days of unloading/hindlimb suspension (HS) and three days HS with HDACs inhibitor LMK-235 (HSLMK) or Tq (HSTq). Treatment with LMK-235 diminished unloading-induced of MAFbx, myogenin (MYOG), ubiquitin and calpain-1 mRNA expression (*p* < 0.05). Tq administration had no effect on the expression of E3-ligases. The mRNA expression of MuRF1 and MAFbx was significantly increased in both HS and HSTq groups (1.5 and 4.0 folds, respectively; *p* < 0.05) when compared with the C group. It is concluded that during three days of muscle unloading: (1) the HDACs 4 and 5 participate in the regulation of MAFbx expression as well as the expression of MYOG, ubiquitin and calpain-1; (2) the inhibition of HDAC 4 has no effect on MAFbx expression. Therefore, HDAC 5 is perhaps more important for the regulation of MAFbx expression than HDAC 4.

## 1. Introduction

There are very few studies focusing on the triggering mechanisms involved in the regulation of protein degradation during the early stages of skeletal muscle unloading. A majority of the previous studies evaluated the role of nuclear transcription factor FoxO phosphorylation in the regulation of expression of the E3 ubiquitin ligases MAFbx and MuRF1 during unloading. Typically, activation of the Akt/mTOR/p70S6k pathway was analyzed. Suppression of this pathway during muscle unloading leads to the dephosphorylation and nuclear translocation of FoxO with subsequent activation of the expression of E3 ubiquitin ligases [1,2]. In previous studies, we documented that during muscle unloading increased expression of E3 ubiquitin ligases was accompanied by the activation of some components of the Akt-FoxO pathway while other components were not changed [3,4]. This suggests that some other pathways can regulate activation of the E3 ubiquitin ligases during unloading. Recent studies suggest that HDACs can directly interact with and regulate the activation of transcription factors [5,6,7].

We examined whether these alternative mechanisms are working via HDACs 4 and 5 to regulate the expression of E3 ubiquitin ligases during unloading. Specifically, we were interested in whether HDACs 4 and 5 modulate the expression/activity of FoxO and MYOG. Both of these transcription factors are known to play a critical role in the activation of E3 ligases during unloading. HDACs 4 and 5 belong to the class IIa HDACs. They promote neurogenic muscle atrophy via the transcriptional repression of Dach2 that is responsible for the inhibition of MYOG-dependent atrophy-induced genes [8,9]. Previous studies reported that activation of E3 ubiquitin ligases and unloading-induced skeletal muscle atrophy were diminished by treatment with the HDACs 4 and 5 inhibitor trichostatin A [10]. Nevertheless, the mechanisms regulating the expression of E3 ubiquitin ligases during trichostatin A treatment are still incompletely understood. Since trichostatin A inhibits both HDACs 4 and 5, it is not clear which of these HDACs has the most significant effect on the expression of E3 ubiquitin ligases during unloading.

In this study, we used HDACs 4 and 5 inhibitor LMK-235 and specific HDAC 4 inhibitor Tasqinimod (Tq) to evaluate the activation of the unloading-induced genes. Currently, there is no specific HDAC 5 inhibitor available. We used three days of unloading time point in our study since at this time there is maximal activation of the E3 ubiquitin ligases MAFbx and MuRF1 in soleus muscle [11,12]. If our hypothesis about different roles of HDACs 4 and 5 in unloading is correct, then the comparison of LMK-235 and Tq inhibitors should reveal which of the two HDACs is the most important for the regulation of MYOG, MAFbx and MuRF1 expression, as well as FoxO phosphorylation.

## 2. Results

### 2.1. Effect of HDAC Inhibitors on the Expression and Activity of HDACs 4 and 5

**Figure 1:**
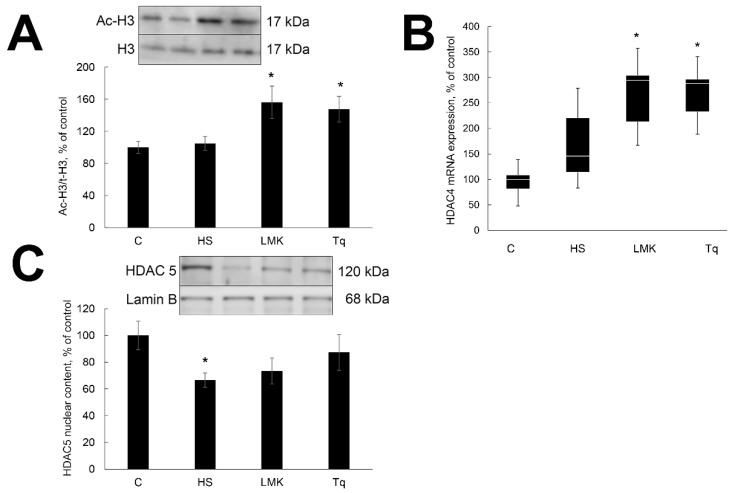
Evaluation of acetylated histone H3 content (**A**), histone deacetylases (HDAC) 4 mRNA expression (**B**) and HDAC 5 nuclear content (**C**) in soleus muscles of nontreated control rats (**C**), rats after 3 days of unloading (HS), 3 days HS with LMK-265 inhibitor (HSLMK) or HS with Tq inhibitor (HSTq). Values are normalized to the levels of total histone H3 (**A**) or Lamin B1 (**C**) in each sample. Levels of HDAC 4 mRNA were normalized to the levels of GAPDH in each sample (**B**). *n* = 8. * indicates a significant difference from the control, *p* < 0.05.

To test whether treatment with LMK-235 and Tq affected the activity of HDACs during three days of unloading we evaluated the content of acetylated histone H3 in HS, HSMLK, Tq and C groups. Acetylation of H3 was significantly higher in the soleus muscle of HSMLK and Tq rats when compared with HS and C rats (Figure 1A). LMK-235 and Tq treatment significantly increased mRNA expression of HDAC 4 when compared with HS and C groups (Figure 1B). The content of the nuclear fraction of HDAC 5 was significantly decreased by unloading (Figure 1C). Treatment with LMK-235 had no significant effect on the nuclear HDAC 5 content, while Tq showed a trend for the increase when compared with the HS group (Figure 1C).

### 2.2. Effect of HDAC Inhibitors on Skeletal Muscle Atrophy

**Figure 2:**
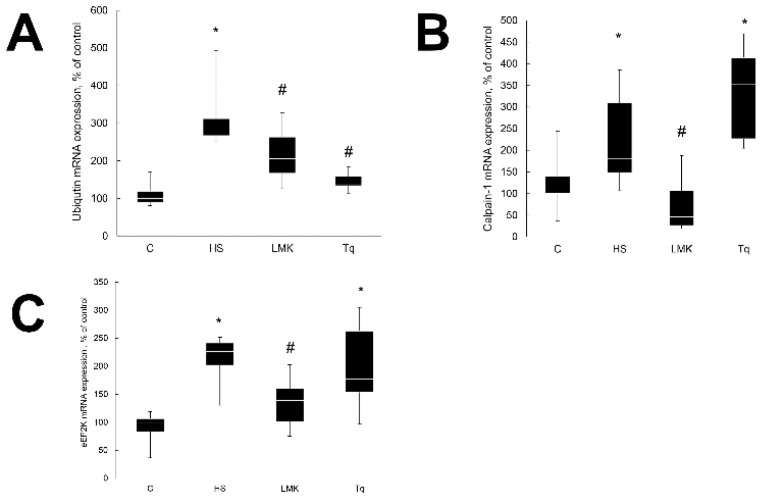
Evaluation of the ubiquitin (**A**), calpain-1 (**B**) and eEF2k (**C**) mRNA expression in soleus muscles of nontreated control rats (C), rats after 3 days of unloading (HS), 3 days HS with LMK-265 inhibitor (HSLMK) or HS with Tq inhibitor (HSTq). Values are normalized to the levels of GAPDH mRNA expression in each sample. *n* = 8. * indicates a significant difference from the control, *p* < 0.05; # indicates a significant difference from the HS, *p* < 0.05.

After three days of unloading soleus muscle mass decreased by approximately 20% in all three groups. In HS rats it was 74.5 ± 3.0 mg (*p* < 0.05), in HSLMK 72.8 ± 3.1 mg (*p* < 0.05), and in HSTq 73.0 ± 2.3 mg (*p* < 0.05), while in C rats it was 93.5 ± 2.4 mg. At the same time, there was a significant difference in the mRNA expression of ubiquitin and calpain-1 between the groups (Figure 2A,B). The expression of ubiquitin was significantly higher in the soleus muscle of HS rats when compared with C rats (Figure 2A). LMK-235 and Tq treatment significantly augmented the unloading-induced ubiquitin expression (Figure 2A). The mRNA expression of ubiquitin was not significantly different in HSMLK and HSTq groups when compared with C.

Calpain-1 is calcium-activated proteinase involved in the degradation of myofibrillar proteins during skeletal muscle atrophy [13]. The expression of calpain-1 was significantly increased after skeletal muscle unloading (Figure 2B).Treatment with LMK-235 blocked the increase of calpain-1 mRNA expression during unloading (Figure 2B). At the same time, treatment with Tq showed a trend for the increase of calpain-1 mRNA expression when compared with the HS group (Figure 2B).

Eukaryotic elongation factor 2 kinase (eEF2k) regulates protein synthesis and protein expression of eEF2k was previously shown to be upregulated by seven days of unloading [14]. In the current study, the mRNA expression of eEF2k was increased by unloading (Figure 2C). Treatment with LMK-235 diminished this increase, while Tq treatment had no statistically significant effect (Figure 2C).

**Figure 3:**
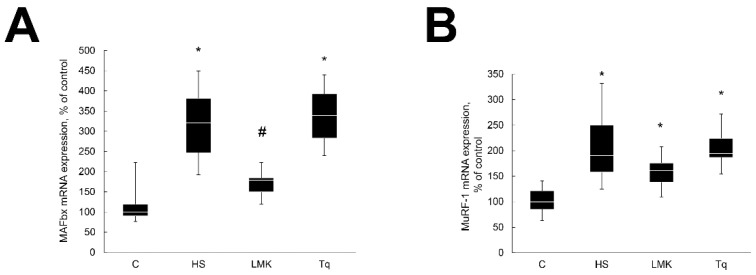
Evaluation of the MAFbx (**A**) and MuRF1 (**B**) mRNA expression in soleus muscles of nontreated control rats (**C**), rats after 3 days of unloading (HS), 3 days HS with LMK-265 inhibitor (HSLMK) or HS with Tq inhibitor (HSTq). Values are normalized to the levels of GAPDH mRNA expression in each sample. *n* = 8. * indicates a significant difference from the control, *p* < 0.05; # indicates a significant difference from the HS, *p* < 0.05.

It was previously reported that the expression of E3 ubiquitin ligases MAFbx and MuRF1 is increased after three days of unloading [15]. In the current study, the mRNA expression of MAFbx and MuRF1 was also significantly increased by unloading (Figure 3). Treatment with LMK-235 blocked the upregulation of MAFbx mRNA expression, while Tq treatment had no effect (Figure 3A). Neither LMK-235 nor Tq treatments had a significant effect on the MuRF1 mRNA expression (Figure 3B).

### 2.3. Effect of HDAC Inhibitors on Transcription Factors Regulating Expression of MAFbx and MuRF1

**Figure 4:**
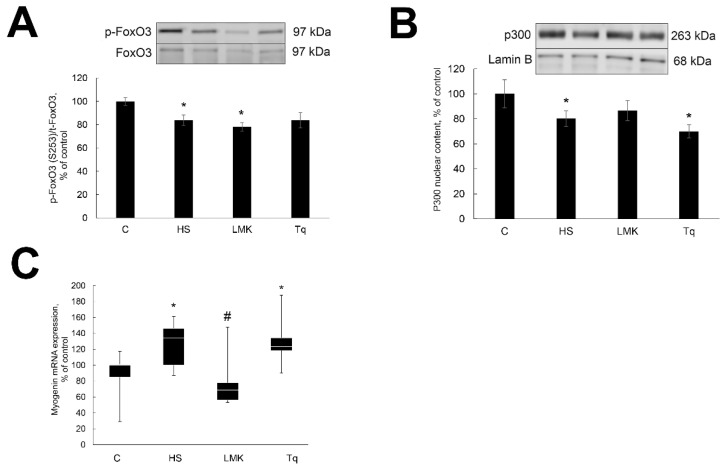
Evaluation of phosphorylated FoxO3 content (**A**), P300 nuclear content (**B**) and MYOG mRNA expression (**C**) in soleus muscles of nontreated control rats (**C**), rats after 3 days of unloading (HS), 3 days HS with LMK-265 inhibitor (HSLMK) or HS with Tq inhibitor (HSTq). Values are normalized to the levels of total FoxO3 protein (**A**) or Lamin B1 (**B**) in each sample. Levels of MYOG mRNA were normalized to the levels of GAPDH in each sample (**C**). *n* = 8. * indicates a significant difference from the control, *p* < 0.05; # indicates a significant difference from the HS, *p* < 0.05.

FoxO3, MYOG and P300 are known regulators of MAFbx and MuRF1 expression during unloading (Bodine and Baehr, 2014). The current study tested whether HDACs 4 and 5 regulate the activity of these transcription factors during unloading. The content of phosphorylated FoxO3 was decreased in the HS group when compared with C (Figure 4A). Treatment with LMK-235 and Tq had no statistically significant effect on the content of phosphorylated FoxO3 during unloading (Figure 4A).

The nuclear content of P300 was decreased after three days of soleus muscle unloading (Figure 4B). Treatment with LMK-235 showed a trend for lessening this increase, while Tq treatment had no statistically significant effect (Figure 4B).

The mRNA expression of MYOG was increased after three days of unloading (Figure 4C). Treatment with LMK-235 blocked this increase (Figure 4C), while Tq treatment had no statistically significant effect (Figure 4C).

## 3. Discussion and Conclusions

Unloading results in skeletal muscle atrophy via activation of protein degradation and decrease of protein synthesis [16,17]. E3 ubiquitin ligases MAFbx and MuRF1 play a significant role in this process. In the current study, we evaluated whether HDACs 4 and 5 are involved in the regulation of unloading-induced processes of muscle atrophy.

There was no significant effect of HDACs 4 and 5 inhibitor LMK-235 or HDAC 4 inhibitor Tq on the unloading-induced soleus muscle atrophy in our study. Three days is a minimal time point for the detection of muscle atrophy during unloading [18]. Previous studies reported attenuation of muscle atrophy with HDACs 4 and 5 inhibitors after 14 days of unloading [10]. It is likely that at the later stages of muscle unloading HDACs 4 and 5 play a larger role in the regulation of muscle atrophy when compared with the early stages of unloading.

The ubiquitin–proteasome pathway regulates the degradation of sarcomeric proteins during muscle atrophy [19]. During unloading the expression of ubiquitin is significantly increased [16]. Treatment with LMK-235 and Tq blocked the unloading-induced increase in the ubiquitin mRNA expression in our study. Calpains are calcium-activated proteinase that can be the first step in the initiation of protein ubiquitination leading to muscle atrophy during unloading. Some data suggest that calpains and ubiquitin–proteasome pathways are not always synchronized during skeletal muscle atrophy [20].

eEF2k regulates protein synthesis during muscle atrophy [21]. eEF2 kinase phosphorylates eEF2k blocking its translocation into the nucleus. This leads to the inhibition of protein synthesis. The expression of eEF2k was significantly increased in HS and HSTq groups suggesting that protein synthesis was decreased in these two groups. Inhibition of HDACs 4 and 5 with LMK-235 diminished eEF2k increase, indicating that impairment of protein synthesis might be reduced.

Treatment with LMK-235 and Tq significantly increased the expression of HDAC 4 during unloading. This might be a feedback response to the inhibition of the HDAC activity and this confirms the activity of LMK-235 and Tq inhibitors in the current study. The content of HDAC 5 in the nucleus was decreased by muscle unloading in our experiments. It was previously reported that protein kinase D 1 (PKD1) creates recognition sites for the phosphorylated 14-3-3 chaperon protein on HDAC 5 [22]. Binding of 14-3-3 to HDAC 5 leads to the translocation of HDAC 5 out of the nucleus. Similar processes might be involved in the decrease of HDAC 5 content in the HS group in our study, but this requires further investigations. It is known that HDACs control the promoter activity of genes coding for E3 ubiquitin ligases [23]. HDAC 5 can directly bind to transcription factor EB (TFEB) and inhibit TFEB-mediated MuRF1 expression [22]. Currently, there is no HDAC 5–specific inhibitor. We used LMK-235 that inhibits both HDACs 4 and 5, as well as HDAC 4-specific inhibitor Tq. The unloading-induced mRNA expression of MAFbx was blocked by LMK-235 but not Tq in our experiments. Moresi and colleagues previously showed that skeletal muscle-specific knockout of HDACs 4 and 5 in mice results in a significant decrease of denervation-induced MuRF1 and MAFbx expression [24]. At the same time, inhibition of HDACs 4 and 5 with trichostatin A during 14 days of muscle unloading diminished upregulation of MuRF1 expression but had no effect on MAFbx expression [10]. Therefore, the data presented in the current study correlate with the previous observations. We showed for the first time that HDACs 4 and 5 regulate MAFbx expression during three days of muscle unloading. Based on our results, HDAC 5 and not HDAC 4 plays the most critical role in the upregulation of MAFbx during unloading.

During muscle denervation, HDACs 4 and 5 regulate MuRF1 and MAFbx expression via the expression of MYOG [24,25,26]. MYOG is a muscle-specific transcription factor involved in the coordination of numerous processes in skeletal muscle, including prolonged muscle immobilization [27]. It was previously reported that MHYOG is upregulated during prolonged muscle immobilization [27]. MYOG can directly bind to MuRF1 and MAFbx promoters and upregulate expression of these genes [24,25]. Blocking MYOG expression decreases denervation-induced MuRF1 and MAFbx expression and prevents muscle atrophy [24]. Our study showed significant upregulation of MYOG in response to unloading. Treatment with LMK-235 blocked unloading-induced upregulation of MYOG, while Tq treatment had no effect. Therefore, HDAC 5 plays a larger role in the MYOG-mediated MAFbx expression during unloading.

In addition to HDACs 4/5–MYOG signaling, several other signaling pathways are known to regulate the expression of MuRF1 and MAFbx during muscle atrophy [26]. For example, PI3K-Akt-mTORC1-FoxOs signaling. FoxO3 can interact with the promoters of MuRF1 [16,28,29] and MAFbx [16] and activate the expression of these genes. Class IIa HDACs can deacetylate and activate FoxO transcription factors [30]. To test the activity of FoxO3 we evaluated its phosphorylation and compared it to the level of MuRF1 and MAFbx expression. FoxO3 phosphorylation was decreased by unloading. Treatment with Tq diminished unloading-induced decrease of FoxO3 phosphorylation, while LMK-235 treatment had no effect. Changes in unloading-induced FoxO3 phosphorylation did not correlate with the level of MuRF1 and MAFbx expression. It is unlikely that FoxO3 phosphorylation is involved in HDACs 4/5-mediated effects on MuRF1 and MAFbx expression in our study.

HDACs play a critical role in the repression of gene transcription by histone deacetylation and increasing chromatin condensation [31,32,33]. We evaluated the acetylation levels of the N-terminal end of histone H3. Unloading-induced histone H3 acetylation was significantly diminished by the treatment with both LMK-235 and Tq inhibitors. In addition to the regulation of gene transcription through the regulation of histone acetylation, histone acetyltransferases (HATs) and HDACs regulate gene transcription via acetylation/deacetylation of transcription factors [31]. It was previously reported that HAT acetylates FoxO3 [34]. A decrease of HAT activity increases FoxO3 transcriptional activity, while the increase of HAT activity interferes with nuclear translocation of FoxO3 and diminishes its transcriptional activity towards target genes [34]. Similarly, the decrease of MAFbx expression during muscle unloading in response to LMK-235 treatment might be mediated by FoxO3 acetylation and not by FoxO3 phosphorylation/dephosphorylation.

FoxO3 transcriptional activity can be also regulated via acetylation by P300 [35]. P300 acetylates FoxO3 diminishing its transcriptional activity and preventing nuclear localization [35]. It was previously reported that a decrease of P300 activity during skeletal muscle denervation leads to the activation of FoxO3 and increased expression of its target gene MAFbx [35]. Our study showed a decrease of P300 nuclear content in HS and HSTq groups, while the P300 nuclear content in the HSLMK group was similar to the control group. The expression of MuRF1 and MAFbx was also the highest in HS and HSTq groups in our study. This supports our hypothesis that the LMK-235-mediated decrease of MAFbx mRNA expression in our experiments might be regulated by the decrease of transcriptional activity of FoxO3 due to its increased acetylation.

In conclusion, the current study showed that after three days of skeletal muscle unloading HDACs 4 and 5 regulate mRNA expression of MYOG, MAFbx, calpain-1 and ubiquitin. Treatment with specific HDAC 4 inhibitor Tq affected only the ubiquitin mRNA expression. This suggests that HDAC 5 plays a more significant role than HDAC 4 during unloading-induced skeletal muscle atrophy.

## 4. Materials and Methods

### 4.1. Ethical Approval

All animal experiments were performed at the Institute of Biomedical Problems, RAS, Russia. All experiments were approved by the Committee on Bioethics of the Russian Academy of Sciences (protocol 448; 03/28/2017). The experiments were performed in accordance with the internationally accepted regulations and rules of biomedical ethics and comply with the principles and regulations described by Grundy [36].

### 4.2. Animal Procedures

Animals were kept at 22 °C in a light-controlled environment (12:12 h light–dark cycle) with unlimited access to water and food. In the first set of experiments we optimized the doses of LMK-235 and Tq required for the inhibition of HDACs 4 and 5 during muscle unloading. Previous studies used LMK-235 to inhibit HDACs 4 and 5, and Tq to inhibit HDAC 4 using mouse models [37] and cell lines [38,39]. These inhibitors readily diffuse into cells. The degree of inhibition of HDACs 4 and 5 with LMK-235 and Tq during unloading can be evaluated based on the acetylation of histones.

To determine the optimal dose of LMK-235, 15 male Wistar rats (3 months old, 180–200 g body weight) were randomly assigned to one of five groups (3 animals/group): nontreated control (C), three days of hindlimb suspension/unloading with (HSLMK) or without (HS) HDACs 4 and 5 inhibitor LMK-235 (#A4494, ApexBio, Boston, MA, USA) in the dose of 10, 20, and 30 mg/kg of body weight per day. LMK-235 was dissolved in 2% solution of DMSO in normal saline solution and 200 µL were injected intraperitoneally. Similarly, to determine the optimal dose of Tq 15 male Wistar rats (three months old, 180-200 g body weight) were randomly assigned to one of five groups (3 animals/group): nontreated control (C), three days of hindlimb suspension/unloading with (HSTq) or without (HS) HDAC 4 inhibitor Tq (#A3860, ApexBio, USA) in the dose of 10, 20 and 30 mg/kg of body weight per day. Tq was delivered orally with a small amount of food. We ensured that each rat consumed the entire small piece of food provided with each treatment. The control animals received an intraperitoneal injection of 2% solution of DMSO in normal saline solution (for LMK-235 control) or equal amounts of similar food without inhibitor (Tq control).

The main experiments were performed after the optimal dose was established for both inhibitors. For the LMK-235 optimal concentration was 20 mg/kg of body weight per day injected intraperitoneally. For the Tq optimal concentration was 10 mg/kg of body weight per day delivered orally with a small amount of food. Fifty-six male Wistar rats (180–200 g body weight) were randomly assigned to one of four groups (8 animals/group): nontreated control (C), three days of hindlimb suspension/unloading with (HSLMK) or without (HS) HDACs 4 and 5 inhibitor LMK-235 and HDACs 4 inhibitor Tq (HSTq).

At the end of the 3-day experiment, rats were euthanized by an overdose of 10% avertin solution (Sigma-Aldrich Corp., St. Louis, MO, USA). Soleus muscle was immediately dissected from each rat, weighed, divided into aliquots, frozen in liquid nitrogen, and stored at −85 °C for the analyses.

### 4.3. Hindlimb Suspension

For the hindlimb suspension, a traction method of noninvasive tail-casting procedure was used [40]. A swivel harness system incorporated into the casting materials and attached to a hook at the top of the cage was used for the suspension. The hook was adjusted in a way that allowed only the forelimbs of the animal to reach the floor of the cage while the hindlimbs were suspended. The body axis of the rats was at a 45 angle to the cage floor. Rats were free to move around the cage using forelimbs to consume food and water.

### 4.4. Protein Extraction and Western Blot Analysis

Total protein extracts were prepared from 400 µg of the frozen soleus muscle. To maintain extract integrity and function Complete Protease Inhibitor Cocktail (#sc-29130, Santa Cruz Biotechnology, Dallas, TX, USA), Phosphatase Inhibitor Cocktail B (#sc-45045, Santa Cruz Biotechnology, Dallas, TX, USA), PMSF (1 mM), aprotinin (10 µg/mL), leupeptin (10 µg/mL), and pepstatin A (10 µg/mL) were used. Cytoplasmic and nuclear fractions were isolated using NE-PER Nuclear and Cytoplasmic Extraction Reagents (Thermo Scientific, Waltham, MA, USA). Quick Start Bradford Protein Assay (Bio-Rad Laboratories, Hercules, CA, USA) was used to quantify protein content. The samples were diluted in Laemmli buffer, run on 10% SDS-PAGE (20 µg/lane), and transferred to a nitrocellulose membrane (Bio-Rad Laboratories, Hercules, CA, USA). After blocking with blocking buffer (5% nonfat milk powder, TBS pH 7.4, and 0.1% Tween-20) the membranes were incubated overnight at 4 °C with the primary antibodies.

We used primary antibodies against total Akt (1:1000; #2920, Cell Signaling Technology, USA) and phosphorylated Akt (Ser 473; 1:1500, 4058, Cell Signaling Technology, USA), total histone H3 (1:500, #9715, Cell Signaling Technology, Danvers, MA, USA) and acetylated histone H3 (1:500, #06-599, Millipore, Burlington, MA, USA), total FoxO3 (1:1000; #2497, Cell Signaling Technology, Danvers, MA, USA) and phosphorylated FoxO3 (Ser 253; 1:1000; #sc-101683, Santa Cruz Biotechnology, Dallas, TX, USA), total HDAC 5 (1:3000, #ab1439, ABCAM, Cambridge, MA, USA), total HAT P300 (1:2000, #ab231010, ABCAM, Cambridge, MA, USA). Blots incubated with antibodies against GAPDH (1:10,000, #G041, Applied Biological Materials Inc., Richmond, BC, Canada) were used for the normalization of the loading of cytoplasmic fractions. Lamin B1 content (1: 1000, #ab16048, ABCAM, Cambridge, MA, USA) was used for the normalization of the loading of nuclear fractions. After three washes (10 min each) with TBS-Tween (TBS and 0.1% Tween-20), the membranes were incubated for one hour at room temperature with horseradish peroxidase-conjugated goat anti-rabbit (1:30,000, #111-035-003, Jackson Immuno Research, West Grove, PA, USA) or goat anti-mouse (1:20,000, #1706516, Bio-Rad, Hercules, CA, USA) secondary antibodies. The membranes were washed again in TBS-Tween three times, incubated with Clarity Western ECL Substrate (Bio-Rad Laboratories, Hercules, CA, USA). All images were analyzed within the linear range. The protein bands were quantified using a C-DiGit Blot Scanner (LI-COR Biotechnology, Lincoln, NE, USA) and Image Studio C-DiGit software. Total protein staining (Ponceau S) was used for the control of loading. The protein expression data for each group are expressed as a percentage of the control group values.

### 4.5. RNA Isolation and Reverse Transcription

Eighty micrograms of frozen soleus muscle were used for isolation of total RNA using RNeasy Micro Kit (Qiagen, Hilden, Germany). RNA samples were treated with proteinase K and DNase I. RNA concentration was evaluated using a NanoPhotometer (Implen GmbH, Munich, Germany). Isolated RNA in aqueous solution was frozen at −85 °C for storage. Reverse transcription was performed by incubating 0.5 µg of RNA, random hexamers d(N)6, dNTPs, RNase inhibitor, and MMLV (Moloney Murine Leukemia Virus) reverse transcriptase (Moscow, Russia) for 60 min at 42 °C.

### 4.6. Quantitative PCR Analysis

One microliter of cDNA was amplified in a 25 µL SYBR Green PCR reaction containing 1× Quantitect SYBR Green Master Mix (Syntol, Moscow, Russia) and 10 pM of each forward and reverse primer. The sequences of the primers used in the current study are presented in Table 1. All primers were synthesized by Syntol (Moscow, Russia). The optimal temperature for each PCR primer pair was used for annealing. The amplification was monitored in real-time using an iQ5 Multicolor Real-Time PCR Detection System (Bio-Rad Laboratories, Hercules, CA, USA). Melting curve analysis was used to confirm the amplification specificity. Relative quantification was performed based on the threshold cycle (CT value) for each PCR sample [41]. Initially, two housekeeping genes were evaluated for normalization: GAPDH and β-actin. Normalizat`1aaw aion of the level of expression of GAPDH and β-actin showed similar results. GAPDH was chosen for the normalization of all quantitative PCR analysis experiments in the current study.

### 4.7. Statistical Analysis

All PCR data are expressed as the median and interquartile range (0.25–0.75). Statistical analysis was performed using the REST 2009 v.2.0.12 (Qiagen, Hilden, Germany) and Origin Pro v.8.0 (OriginLab Corp., Northampton, MA, USA) programs. All Western blot data are expressed as means ± SE. Significant differences between groups were statistically analyzed using two-way ANOVA followed by Tukey’s test. When normality testing failed, data were analyzed by nonparametric methods (Kruskal–Wallis ANOVA followed by Dunnett’s test). Differences with values of *p* < 0.05 were considered statistically significant.

## Figures and Tables

**Table 1:** Primers used for QRT-PCR study.

Gene	Forward Primer	Reverse Primer
**β-actin**	5′- TCATGAAGTGTGACGTTGACATCC -3′	5′- GTAAAACGCAGCTCAGTAACAGTC -3′
**Calpain-1**	5′- CATGGCTAAGAGCAGGAAGG -3′	5′- CGAAGTCTGCAGGTCTAGGG -3′
**eEF2k**	5′- AGAAGCTGGTGACAGGCAGT -3′	5′- GGGTTCTTGTCCAGTCCAAA -3′
**GAPDH**	5′- ACGGCAAGTTCAACGGCACAGTCAA -3′	5′- GCTTTCCAGAGGGGCCATCCACA -3′
**HDAC 4**	5′- CTACAACCACCCTGTCTTGG -3′	5′-ATGCGGAGTCTGTAACATCC-3′
**MAFbx**	5′- CTACGATGTTGCAGCCAAGA -3′	5′- GGCAGTCGAGAAGTCCAGTC -3′
**MuRF1**	5′- GCCAATTTGGTGCTTTTTGT -3′	5′- AAATTCAGTCCTCTCCCCGT -3′
**MYOG**	5′- ACTCCCTTACGTCCATCGTG -3′	5′- CAGGACAGCCCCACTTAAAA -3′
**Ubiquitin**	5′- CACCAAGAAGGTCAAACAGGA -3′	5′- GCAAGAACTTTATTCAAAGTGCAA -3′
